# Direct Effects of Clinically Relevant Antibiotics on Mitochondrial Respiration

**DOI:** 10.3390/ijms26115379

**Published:** 2025-06-04

**Authors:** Judith Sailer, Sabine Schmitt, Hans Zischka, Erich Gnaiger

**Affiliations:** 1 Institute of Toxicology and Environmental Hygiene, TUM School of Medicine and Health, Technical University Munich (TUM), 80802 Munich, Germany; judith.sailer@tum.de; 2 Oroboros Instruments, Schoepfstrasse 18, 6020 Innsbruck, Austria; sabine.schmitt@oroboros.at (S.S.); erich.gnaiger@oroboros.at (E.G.); 3 Institute of Molecular Toxicology and Pharmacology, Helmholtz Munich, 85764 Neuherberg, Germany

**Keywords:** antibiotics, mitochondria, high-resolution respirometry

## Abstract

Antibiotics are indispensable in medical patient care, yet they may elicit off-target effects, particularly by affecting mitochondrial function. This study investigates three commonly used antibiotics, gentamicin, ciprofloxacin, and amoxicillin, for their direct effects on mitochondrial respiration and membrane potential. Using high-resolution respirometry, we show that gentamicin and ciprofloxacin markedly increase mitochondrial leak respiration in permeabilized human embryonic kidney cells, suggesting alterations in the mitochondrial inner membrane. This is supported by a gentamicin-induced decrease in mitochondrial membrane potential. Especially gentamicin, but also ciprofloxacin, dose- and time-dependently inhibit oxidative phosphorylation and the mitochondrial electron transfer capacity, pronouncedly in the NADH-linked but also in the succinate-linked pathway. Furthermore, gentamicin decreases Complex IV (CIV) activity in a time-dependent fashion. In contrast, amoxicillin has no significant effect on mitochondrial respiration. These findings emphasize the importance of evaluating the potential direct toxicity of antibiotics on mitochondria, as they are most critical off-target sites. High-resolution respirometry provides a powerful approach to characterize such effects early in the drug development process.

## 1. Introduction

In 1928, Alexander Fleming discovered the antibiotic penicillin [1], ushering a new era in combating bacterial infections. Contrary to their life-saving benefits, including reduced mortality and enabling complex surgeries [2,3,4,5], antibiotics are, however, also potentially associated with severe side effects. Their most prominent and well-known unwanted effect is a negative impact on the microbiome [6]. More recently, increasing attention has focused on mitochondrial (mt) dysfunction induced by antibiotics [7,8,9,10]. The latter may plausibly be due to the evolutionary origin of mitochondria as bacterial endosymbionts, as evidenced, e.g., by shared genomic and ribosomal features [11,12].

While many studies have reported mid- to long-term antibiotic-induced mt damage upon prolonged exposure [13,14,15], few have investigated acute or direct mt impairing effects [10,16]. Notably, the antibiotics gentamicin, ciprofloxacin, and the combination of amoxicillin with clavulanate that act against a broad spectrum of pathogens, have been implicated in hepatotoxicity and nephrotoxicity [17,18,19,20].

The aminoglycoside gentamicin impairs bacterial protein synthesis by binding to their 16SRNA in the 30S ribosomal subunit [21,22,23]. It has a high clinical efficacy but carries risks of oto- and nephrotoxicity [24]. Some reports postulate a correlation between an increased concentration of reactive oxygen species (ROS) in mitochondria and gentamicin-induced ototoxicity [25,26]. Others report that mt dysfunction leads to increased ROS production, contributing to the development of nephrotoxicity in gentamicin-treated rats [27,28,29]. Moreover, the mitochondrial membrane potential (mtMP) is depolarized in isolated rat liver and kidney mitochondria, and sensory hair cells, following treatment with concentrations of more than 1 mM (0.5 mg/mL) gentamicin [16].

The fluoroquinolone ciprofloxacin inhibits the bacterial gyrase and topoisomerase IV [30]. It is effective against a broad spectrum of bacteria, but adverse effects include optic neuropathy, arrhythmias, and rare cases of retinal detachment, tendinopathy, and tendon rupture, thereby being associated with a black-box warning by the FDA [31]. This limits its application to severe infections. In vitro studies show that ciprofloxacin may depolarize the mtMP and trigger apoptosis by the release of cytochrome *c* [10,15,32].

Amoxicillin, a beta-lactam antibiotic, inhibits bacterial cell wall synthesis [33]. Due to an increasing resistance of bacteria against beta-lactam antibiotics, it is mainly applied together with the beta-lactamase inhibitor clavulanate [34,35,36]. Although it is generally well tolerated [37], especially in monotherapy, its combination with clavulanate is frequently associated with drug-induced liver injury (DILI) [19,38,39]. Reports about effects on mitochondria are rare, but the combination has been linked to opening of the mitochondrial permeability transition pore in isolated rat liver mitochondria [10].

Despite numerous reports highlighting unwanted effects of antibiotics on mt function, systematic investigations on their direct impact on these key metabolic organelles remain rare. Establishing such tests is essential for uncovering potential toxic effects on mitochondria—preferably already at an early stage of drug development—and should become a central part of drug screening. Addressing this gap, the present study aims to investigate the direct effects of the clinically relevant antibiotics gentamicin, ciprofloxacin, and amoxicillin on mitochondrial respiration in permeabilized human embryonic kidney (HEK) 293T cells using high-resolution respirometry (HRR). The HEK 293T cells were chosen in the light of antibiotic-provoked nephrotoxicity. Established protocols for permeabilization of their plasma membrane allows the study of direct toxicity of antibiotics on mitochondria, distinct from secondary effects mediated by cellular metabolism. Mitochondrial respiration is a sensitive marker to assess such potential toxic effects. HRR is the state-of-the-art method to study mitochondrial respiration, and substrate-uncoupler-inhibitor titration protocols (SUIT, Oroboros Instruments) are designed to elaborate respiratory control in a sequence of coupling and pathway control states [40].

Our findings reveal that gentamicin and ciprofloxacin directly increase leak respiration and impair OXPHOS and ET capacity, with gentamicin also reducing mtMP and Complex IV activity.

## 2. Results

The direct, dose- and/or time-dependent effects of the antibiotics gentamicin, ciprofloxacin, and amoxicillin on mitochondrial respiration were examined in HEK 293T cells. Routine respiration was measured prior to antibiotic addition, with a consistent O_2_ flow per cell of 23.03 ± 4.09 amol∙s^−1^∙x^−1^ (*N* = 23, Supplementary Figure S1 and Table S1) being recorded across all experiments. Upon cell membrane permeabilization with digitonin and the addition of pyruvate and malate, leak respiration in the N-pathway was determined to be 6.12 ± 1.5 amol∙s^−1^∙x^−1^ across all trials. The effect of the antibiotics was investigated either in the leak state in the N-pathway (N*_L_*, depicted in red), OXPHOS state in the N-pathway or convergent NS-pathways (N*_P_* or NS*_P_*, depicted in green), and ET state in the convergent NS-pathway or S-pathway (NS*_E_* or S*_E_*, depicted in blue), or in subsequently interrogated OXPHOS or ET states in a variety of pathway states.

**Figure 1:**
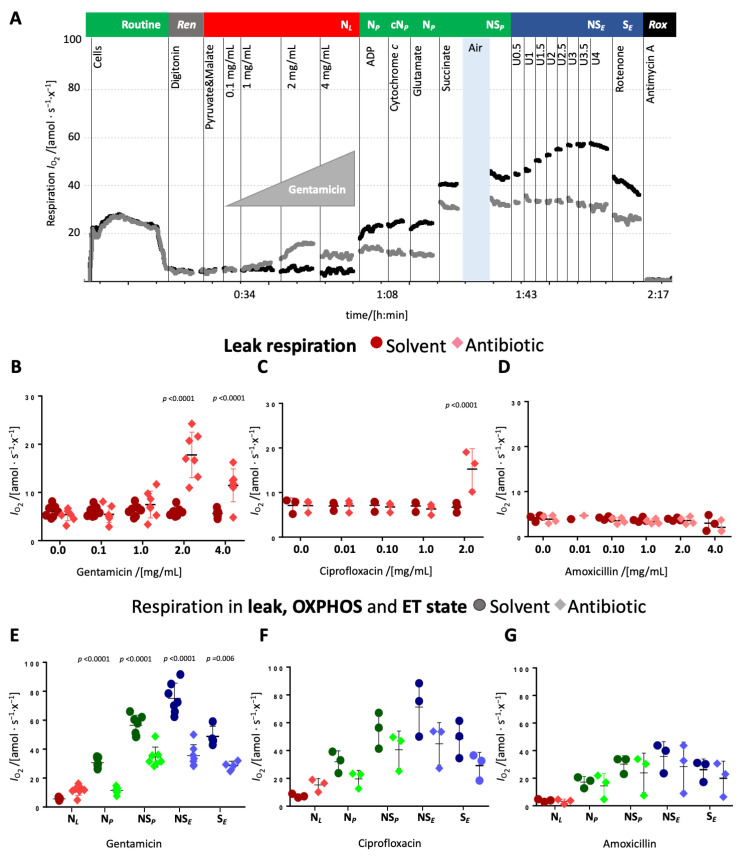
Effect of antibiotics on N-linked leak respiration and subsequent pathway and coupling control states. (**A**) Representative traces of oxygen flow per cell [amol·s^−1^·x^−1^] using the protocol SUIT-008 D025 (37 °C, MiR05) with titration of gentamicin (grey trace) and its solvent (black trace) in the leak state. Sequential titrations are indicated by initial experimental concentrations and respiratory rates: Cells 1 million/mL; routine respiration of living cells. Digitonin 10 µg/mL; residual endogenous substrates *ren*. Pyruvate and Malate 5 and 2 mM, respectively; N-pathway leak respiration N*_L_*. Gentamicin (0.1 to 4 mg/mL, grey trace) or solvent control (black trace). ADP 2.5 mM; N-pathway OXPHOS capacity N*_P_*. Cytochrome *c* 10 µM; Nc*_P_*. Glutamate 10 mM; N*_P_*, Succinate 10 mM; NS-pathway OXPHOS capacity, NS*_P_*. U CCCP titrations (0.5 µM steps) to optimum uncoupler concentration 4 µM; NS-pathway ET capacity NS*_E_*; Rotenone 0.5 mM; S-pathway ET capacity S*_E_*. Antimycin A 2.5 µM; residual oxygen consumption *rox*. (**B**–**D**) O_2_ flow per cell [amol∙s^−1^∙x^−1^] in leak state upon titration of gentamicin, ciprofloxacin, or amoxicillin (diamonds) or respective solvent controls (circles). (**E**–**G**) O_2_ flow per cell [amol∙s^−1^∙x^−1^] in different coupling and pathway control states upon titration of gentamicin, ciprofloxacin, or amoxicillin (diamonds) or respective solvent controls (circles) in leak state. Each data point represents a biological replicate (*N* = 3 to 4). Medians are depicted as stripes.

**Titrations of antibiotics in the leak state.** Upon titration in the leak state, gentamicin increased leak respiration modestly at 1 mg/mL but around threefold at 2 mg/mL and slightly decreased it at 4 mg/mL (Figure 1B and Supplementary Table S2). Ciprofloxacin significantly elevated leak respiration at 2 mg/mL (Figure 1C and Supplementary Table S3), while amoxicillin exerted no detectable effect (Figure 1D and Supplementary Table S3). These changes suggest that gentamicin and ciprofloxacin impair the integrity of the mt inner membrane. Gentamicin and ciprofloxacin inhibited N-pathway OXPHOS capacity, whereas amoxicillin again showed no effect (Figure 1E–G). Cytochrome *c* addition did not increase respiration in any condition, indicating preserved mt outer membrane integrity (Figure 1A and Supplementary Table S3, Supplementary Figure S2). Subsequent glutamate addition did not further stimulate respiration (Supplementary Tables S3–S8), neither in the controls nor in the antibiotic-treated cells. The addition of succinate caused a comparable increase in oxygen flow in treated and control cells (Figure 1A,E–G). However, NS-pathway OXPHOS capacity remained lower in gentamicin- and ciprofloxacin-treated cells compared to their respective controls (Figure 1A,E–G). HEK 293T cells typically exhibit an *E−P* excess capacity (i.e., ET capacity > OXPHOS capacity), as evidenced by the stepwise increase in respiration upon uncoupler titration in both control and amoxicillin-treated cells. In gentamicin- and ciprofloxacin-treated cells, uncoupler titration did not have any effect and the NS-linked ET capacity was comparable to NS-linked OXPHOS capacity, implying an inhibition of the electron transfer system (ETS) (Figure 1E–G). Upon rotenone addition, which inhibits Complex I, respiration decreased in control, ciprofloxacin-, and amoxicillin-treated groups but remained largely unchanged in gentamicin-treated cells, pointing towards a nearly complete inhibition of the N-pathway by gentamicin. In both, ciprofloxacin- and gentamicin-treated cells, ET capacity in the S-pathway was slightly lower compared to the respective solvent control suggesting that these antibiotics also impair the S-pathway.

The highest concentration of antibiotics was used to assess their influence on all subsequent coupling and pathway control states after their initial addition in the leak state. Subsequently, a titration of antibiotics was performed during OXPHOS state to examine dose-dependent effects on OXPHOS capacity.

**Titrations of antibiotics in the N-linked OXPHOS state.** The antibiotics or respective solvent controls were titrated to the permeabilized cells in OXPHOS supported by substrates feeding electrons into the N-pathway and saturating concentrations of ADP (N*_P_*).

**Figure 2:**
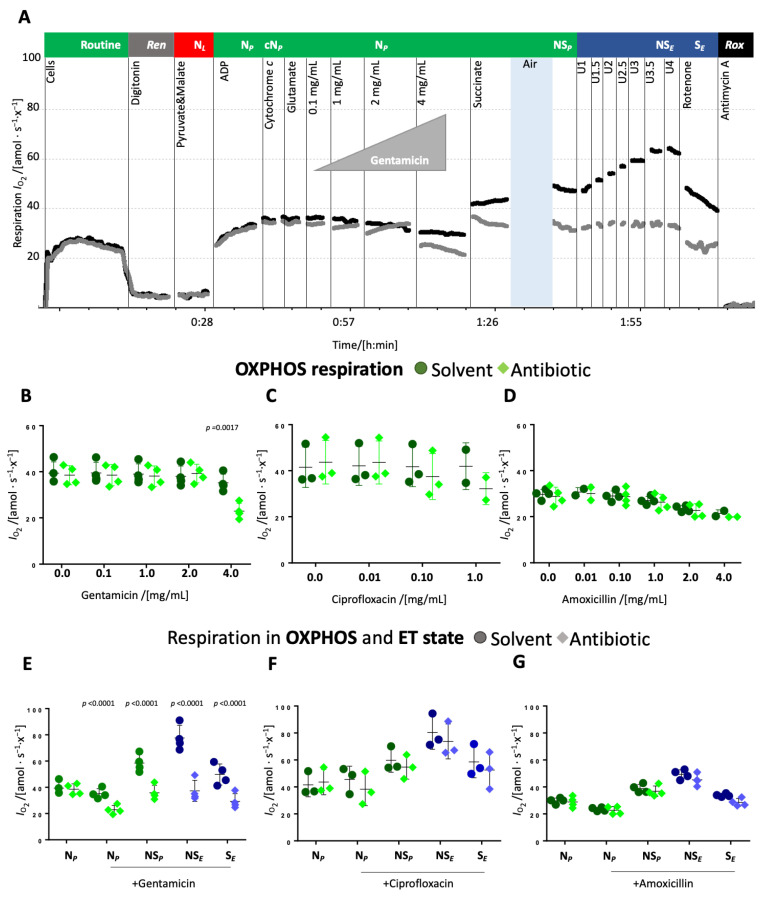
Effect of antibiotics on N-linked OXPHOS capacity and subsequent pathway and coupling control states. (**A**) Representative traces of O_2_ flow per cell [amol·s^−1^·x^−1^] using the protocol SUIT-008_D025 (37 °C, MiR05) with titration of gentamicin (grey trace) and its solvent (black trace) in N-linked OXPHOS state. Sequence of respiratory states (titrations and rates): see Figure 1, gentamicin or solvent alone were added after the addition of ADP. (**B**–**D**) OXPHOS capacity in the N-pathway upon titration of gentamicin (**B**), ciprofloxacin (**C**), or amoxicillin (**D**) (diamonds) or respective solvent control (circles). (**E**–**G**) OXPHOS and ET capacity in different pathway control states after titration of the antibiotic (diamonds) or solvent (circles) in the N-linked OXPHOS state, the underline marks the respective antibiotic addition. Each data point represents a biological replicate (*N* = 2 to 4). Medians are depicted as stripes.

The addition of up to 2 mg/mL gentamicin in the N-linked OXPHOS state did not affect respiration whereas 4 mg/mL gentamicin significantly inhibited N-linked OXPHOS capacity compared to the solvent control (Figure 2A,B and Supplementary Table S4). Ciprofloxacin slightly decreased respiration at a concentration of 0.1 mg/mL and more significantly at a concentration of 1 mg/mL (Figure 2C and Supplementary Table S4). Amoxicillin had no significant effect (Figure 2D and Supplementary Table S4). Interestingly, solvents used for ciprofloxacin and amoxicillin slightly reduced respiration upon repeated titrations. Upon the addition of succinate, respiration increased similarly in controls and ciprofloxacin- and amoxicillin-treated cells (Figure 2F,G), whereas in the presence of gentamicin, respiration increased less than in the solvent control. (Figure 2E). Hence, ET capacity in the NS-pathway was comparable between control and amoxicillin-treated cells (Figure 2G), slightly inhibited in ciprofloxacin-treated cells (Figure 2F), and strongly inhibited in gentamicin-treated cells (Figure 2E). S-pathway ET capacity, measured after the addition of rotenone, was inhibited in gentamicin-treated cells, even though to a lower extent than NS-pathway ET capacity (Figure 2E). In ciprofloxacin- and amoxicillin-treated cells, a slight inhibition of the S-linked ET capacity was observed, comparable to the inhibition of NS-pathway ET capacity (Figure 2F,G).

Taken together, gentamicin and ciprofloxacin showed a dose-dependent effect on N-linked respiration, both the leak and OXPHOS state. In a next step, therefore, we investigated potential dose-dependent effects on the NS-pathway with convergent electron flow into the Q-junction through CI&II.

**Titrations of antibiotics in the NS-linked OXPHOS state.** The antibiotics or solvent controls were titrated to the permeabilized cells in OXPHOS supported by substrates providing electrons via the N- and S-pathway and saturating concentrations of ADP (NS*_P_*).

**Figure 3:**
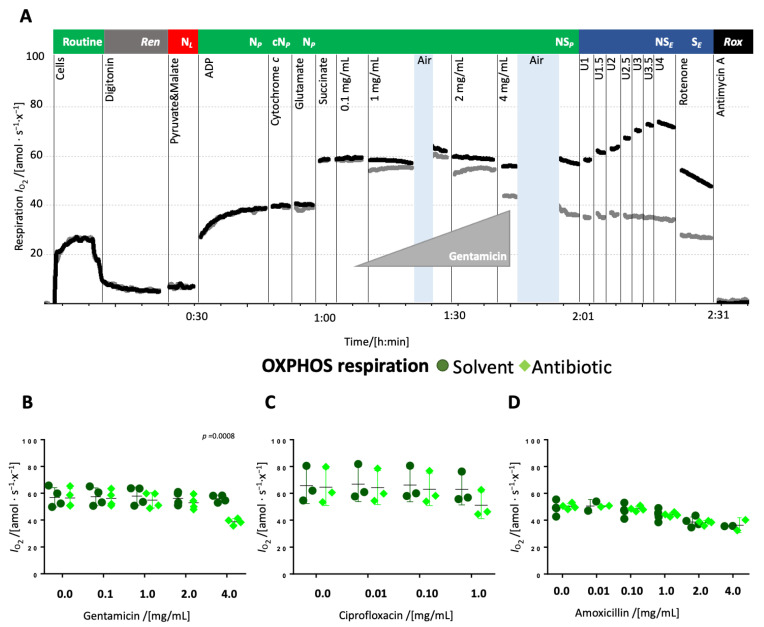
Effect of antibiotics on NS-linked OXPHOS capacity and subsequent pathway and coupling control states. (**A**) Representative trace of the protocol SUIT-008_D025 (37 °C, MiR05) with titration of gentamicin (grey trace) or the solvent (black trace) in NS-linked OXPHOS state, after the addition of succinate. O_2_ flow per cell [amol·s^−1^·x^−1^] with 2·10^6^ HEK 293T cells in the 2 mL chamber. Sequence of respiratory states (titrations and rates): see Figure 1. (**B**–**D**) NS-pathway OXPHOS capacity upon titration of gentamicin (**B**), ciprofloxacin (**C**), or amoxicillin (**D**) (diamonds) or respective solvent control (circles). Each data point represents a biological replicate (*N* = 2 to 4). Medians are depicted as stripes.

**Figure 4:**
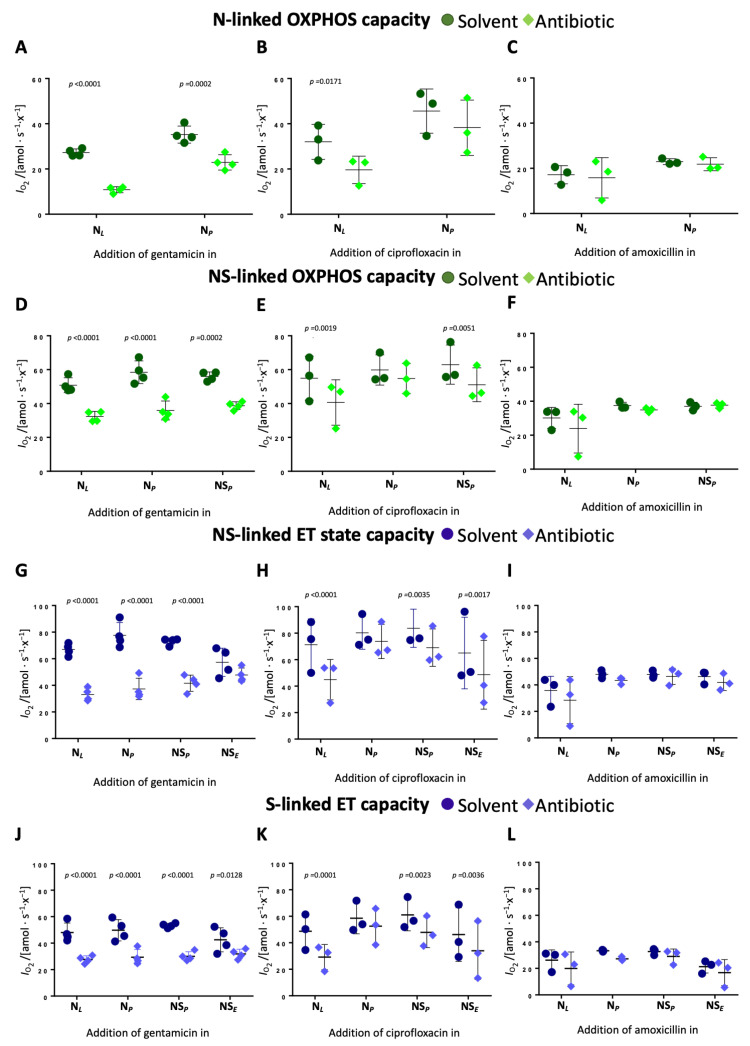
Time-dependent effects on OXPHOS and ET capacity in different pathway control states. The antibiotics gentamicin, ciprofloxacin, and amoxicillin (diamonds) or respective solvents (circles) were titrated at different time points of the protocols and the time-dependent effect was evaluated for (**A**–**C**) N-linked OXPHOS capacity, (**D**–**F**) NS-linked OXPHOS capacity, (**G**–**I**) NS-linked ET capacity, and (**J**–**L**) S-linked ET capacity. Each data point represents a biological replicate (*N* = 3 to 4). Medians are depicted as stripes.

The addition of 4 mg/mL gentamicin reduced respiration in the NS-linked OXPHOS state by approximately 30% whereas lower concentrations had no effect (Figure 3A, Supplementary Table S4). Ciprofloxacin caused merely a mild decrease, while amoxicillin exerted no effect (Figure 3B,C, Supplementary Table S5). NS-pathway ET capacity was impaired in gentamicin-treated samples, but remained unaffected in ciprofloxacin or amoxicillin-treated samples. In gentamicin-treated cells, S-pathway ET capacity was nearly equivalent to the NS-pathway, suggesting near-complete inhibition of the N-pathway. In contrast, NS-pathway ET capacity was higher than in the S-pathway in all other groups (Figure 4D–F, Supplementary Table S5).

The dose-dependent effect of the antibiotics on ET capacity was tested when titrated in the ET state (Supplementary Figure S2). However, the prolonged exposure to the high uncoupler concentration caused a decline in respiration in the solvent controls, thereby complicating the interpretation of the antibiotic’s effect. Nonetheless, gentamicin and ciprofloxacin appeared to reduce S-pathway ET capacity under these conditions.

The previous results demonstrate that gentamicin and ciprofloxacin inhibit N- and NS-linked OXPHOS capacity and NS- and S-linked ET capacity to a notable extent in a dose-dependent manner. In the next step, we examined the time-dependent effects of the antibiotics on OXPHOS and ET capacity.

**Time-dependent effects of gentamicin and ciprofloxacin on OXPHOS and ET capacity.** Time-dependent effects were evaluated by comparing the impact on each respiratory state when antibiotics were added at different stages of the SUIT protocol. Gentamicin reduced N-pathway OXPHOS capacity by 60% when added in leak but only by 35% when added in OXPHOS (Figure 4A, Supplementary Table S6). In the NS-pathway, the degree of inhibition was similar regardless of the time point of addition (approx. 35–38%) (Figure 4D, Supplementary Table S6). Gentamicin reduced NS-pathway ET capacity by 50% when added in the leak state and N-linked OXPHOS state, by 40% when added in the NS-linked OXPHOS state, and had minimal effect when added in the ET state (Figure 4G, Supplementary Table S6). In the S-pathway, gentamicin induced a consistent 40% reduction of ET capacity when added in the N-linked leak and OXPHOS state, and NS-linked OXPHOS state (Figure 4D, Supplementary Table S6).

Ciprofloxacin showed a comparable although slightly less pronounced time-dependent inhibition. N-pathway OXPHOS capacity dropped by 39% when ciprofloxacin was added in leak but only by 16% when added in OXPHOS (Figure 4B, Supplementary Table S6). Ciprofloxacin inhibited NS-pathway OXPHOS capacity by 26% when added in leak and by 18% when added in N- or NS-linked OXPHOS (Figure 4E, Supplementary Table S6). NS-pathway ET capacity showed a 37% reduction when ciprofloxacin was added in leak and a 25% reduction upon addition of ciprofloxacin in the ET state (Figure 4H, Supplementary Table S6). The slight inhibition of S-linked ET capacity was independent of the time point of ciprofloxacin addition. These findings indicate that both antibiotics exhibit time-dependent inhibitory effects on mitochondrial respiration, with earlier exposure resulting in a more pronounced inhibition. Amoxicillin had no measurable time-dependent effects across any respiratory state (Figure 4C,F,I).

The inhibition of the NS- and the S-pathway ET capacities might be associated with an inhibition of the electron transfer system downstream of the Q-junction, e.g., CIV. We therefore determined the effect of the antibiotics on CIV activity.

**Gentamicin inhibits Complex IV activity.** CIV activity was assessed at the end of the protocol after uncoupler titration and antimycin A addition as described in the method section. The chemical background was determined by the addition of sodium azide.

**Figure 5:**
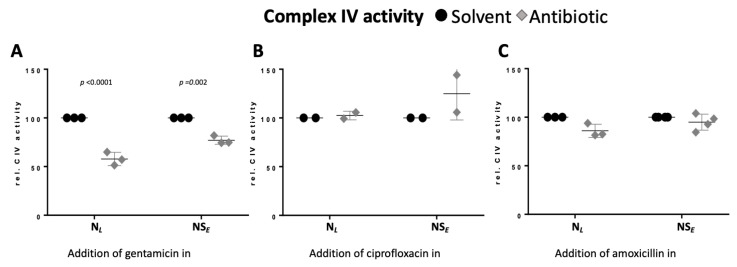
Gentamicin inhibits Complex IV activity. (**A**–**C**) Relative Complex IV activity after the addition of (**A**) gentamicin, (**B**) ciprofloxacin, and (**C**) amoxicillin (diamonds) or solvent control (circles) in N-linked leak state or NS-linked ET state. The respective solvent control was used for normalization. Each data point represents a biological replicate (*N* = 2 to 4). Medians are depicted as stripes.

Gentamicin reduced CIV activity by 42% when added in leak state and by 23% when added in NS-linked ET state, indicating a time-dependent inhibitory effect (Figure 5A, Supplementary Table S7). Ciprofloxacin and amoxicillin exerted no significant effect, although a slight decrease was observed upon the addition of amoxicillin (Figure 5B,C).

**Gentamicin depolarized the mitochondrial membrane potential.** The pronounced effect of gentamicin and ciprofloxacin on leak respiration in HEK 293T cells suggested an impairment of the mt inner membrane integrity which could affect the mitochondrial membrane potential (mtMP). MtMP was measured concurrently with oxygen consumption upon gentamicin addition in the leak state. Similar experiments with ciprofloxacin were precluded due to an interference with the fluorescence signal.

**Figure 6:**
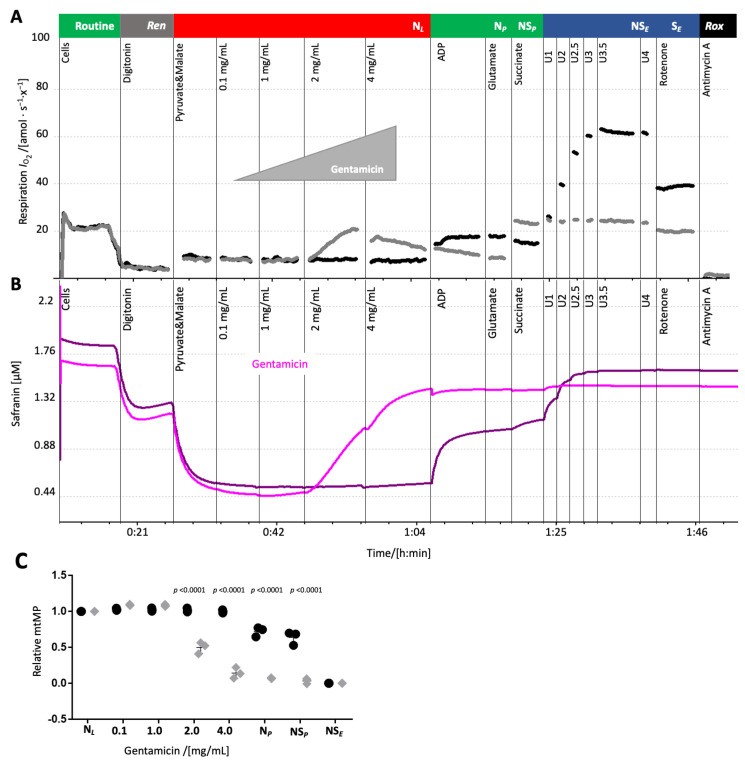
Gentamicin depolarized the mitochondrial membrane potential in HEK 293T cells. Representative trace of (**A**) mitochondrial respiration and (**B**) mtMP in the presence of 2 µM safranin and titration of gentamicin (grey/purple trace) or its solvent (black/magenta trace) in the leak state. Sequence of respiratory states (titrations and rates): see Figure 1 except of cyt *c*. (**C**) Relative changes in mitochondrial membrane potential upon the addition of increasing concentrations of gentamicin (diamonds) or solvent control (circles) in leak state, normalized to the membrane potential in leak state before the addition of gentamicin/solvent. Each data point represents a biological replicate (*N* = 3).

Gentamicin titration in the leak state increased oxygen consumption and simultaneously depolarized the mtMP, shown by the elevated safranin-related fluorescence signal (Figure 6). The solvent (water) exerted no influence on both oxygen consumption and mtMP (Figure 6A,C, Supplementary Table S8). Addition of the uncoupler CCCP did not increase the safranin-related fluorescence signal further, suggesting that the highest concentration of gentamicin induced a collapse of the mtMP.

## 3. Discussion

Despite the molecular similarities between mitochondria and bacteria, the direct impact of antibiotics on mitochondrial function is rarely assessed during the early stages of drug development. As a result, adverse effects such as nephrotoxicity and ototoxicity—potentially linked to mitochondrial dysfunction—may remain undetected until later stages of clinical evaluation.

The present study demonstrates the capacity of high-resolution respirometry (HRR) to detect several direct, dose- and time-dependent effects on mitochondrial function within a single protocol. Using permeabilized HEK 293T cells, we assessed the effect of clinically relevant antibiotics from three different classes and found that gentamicin and ciprofloxacin exert different pronounced effects on mitochondrial function.

**Gentamicin**

Gentamicin significantly increased leak respiration and caused a collapse of the mitochondrial membrane potential, indicating dyscoupling of the electron transfer system from ATP synthesis (Figure 1B and Figure 6), supporting the findings of a previous study [16]. The gentamicin concentrations used herein (e.g., 1 mg/mL, ~2 mM) exceeded therapeutic serum peaks but are relevant considering its pronounced renal accumulation (reported up to 540 µg/g [41], ~1.13 mM). We observed a direct, albeit slight, induction of the leak state at 1 mg/mL.

In addition, gentamicin decreased dose-dependently the N- and the NS-pathway OXPHOS capacity (Figure 2B and Figure 3B). The greater inhibition observed in the N-pathway compared to the NS-pathway points towards a stronger effect on the N- than S-pathway. This interpretation is further supported by the more substantial inhibition of the NS-pathway compared to the S-pathway ET capacity (Figure 1, Figure 2, Figure 3 and Figure 4), and the minimal response to rotenone addition (Figure 1A, Figure 2A, Figure 3A). Even though an impairment of the N-pathway must not necessarily be attributed to a dysfunction of CI, a CI defect is in line with previous studies. These reported gentamicin’s inhibitory effect on CI, although primarily through in vivo studies with extended incubation periods that utilized spectrophotometric measurements of enzyme activity [42,43,44,45]. Additionally, our results demonstrate a time-dependent inhibitory effect of gentamicin on the N-pathway within a short timeframe (Figure 4A,D,G), which plausibly is not attributable to an impaired replication or protein assembly machinery.

S-linked ET capacity was lower in gentamicin-treated samples than in controls (Figure 1 and Figure 4D), indicating that gentamicin exerts an inhibitory effect on the S-pathway which was, however, not time-dependent. This is in line with a previous study showing an inhibitory effect on CII activity [16].

As both the N- and NS-pathway are affected by gentamicin, this points towards an impairment of electron flow downstream of the Q-junction. Indeed, CIII inhibition was reported [16], but our results additionally demonstrate that gentamicin reduced Complex IV activity in a time-dependent manner (Figure 5A). The residual Complex IV activity remained higher than NS-pathway ET capacity, suggesting that Complex IV inhibition might not directly compromise mitochondrial respiration in vivo (Figure 5A). However, the time-dependent nature of this effect suggests that prolonged gentamicin exposure exerts more pronounced effects on Complex IV function. Inhibition of Complex IV activity by aminoglycosides in isolated mitochondria from bovine heart was reported by Kalghatgi et al. (2013) using spectrophotometry [8]. In contrast, gentamicin did not affect the Complex IV activity in young adolescent mice [46]. This discrepancy might be explained by the poor blood–brain barrier permeability of gentamicin, as the mouse study used brain cortex homogenate from treated animals [47].

The protracted renal accumulation of gentamicin, resulting in sustained high concentrations within nephrotic tissue, directly contributes to its established nephrotoxicity by targeting mitochondrial respiratory Complexes [48]. The proposed mechanism involves a synergistic assault on mitochondrial function: inhibition of key respiratory Complexes (I, IV, and possibly ATP synthase), alongside increased membrane permeability and dissipation of the mitochondrial membrane potential. These combined insults impair electron transport capacity, potentially culminating in cellular ATP depletion and subsequent cell death.

**Ciprofloxacin**

The fluoroquinone ciprofloxacin, like gentamicin, dyscoupled electron transfer from the phosphorylation system as evidenced by increased leak respiration. Despite the use of a lower maximum concentration (2 mg/mL compared to 4 mg/mL) due to solubility limitations, ciprofloxacin showed a dyscoupling effect (>2-fold) comparable to gentamicin. An increase in leak respiration occurred at a concentration of 1 mg/mL, which is significantly higher than therapeutic serum peaks (<0.005 mg/mL), suggesting that these effects may only be relevant in the case of single high-dose injections or overdose scenarios [49,50,51]. Measurement of mt membrane potential in the presence of ciprofloxacin was not possible due to interference with the indicator dye (safranin). Ciprofloxacin also inhibited N- and NS-pathway OXPHOS capacity and reduced NS-pathway ET capacity in a time- and dose-dependent manner (Figure 2C,F, Figure 3C and Figure 4B,E,H).

At the applied concentration, ciprofloxacin showed no significant direct effect on Complex IV activity.

The results of our study support the results of previous studies using different methodologies. Kalghatgi et al. (2013) demonstrated inhibitory effects of ciprofloxacin on Complexes I and III in various mammalian cell lines using enzymatic assays, along with mitochondrial membrane potential depolarization, confirming antibiotic-induced mitochondrial dysfunction [8]. A loss of mitochondrial membrane potential was suggested in ciprofloxacin-treated cells upon prolonged incubation (24–72 h) [15]. Overnight exposure to ciprofloxacin increased routine respiration in MIO-M1 cells, with membrane potential loss attributed to ROS overproduction [52]. The cytotoxicity of ciprofloxacin, much like that of gentamicin, is most probably a result of several interconnected mitochondrial dysfunctions. These include the inhibition of oxidative phosphorylation, a concurrent loss of membrane potential, and diminished electron transport capacity, collectively plunging the cell into a bioenergetic crisis that directly underlies its cytotoxic impact. Collectively, these findings highlight the importance of considering mitochondrial toxicity when evaluating the safety profile of ciprofloxacin, particularly in clinical scenarios where drug accumulation might exceed standard therapeutic concentrations.

**Amoxicillin**

Amoxicillin is rarely administered as monotherapy in current clinical practice due to widespread bacterial resistance to beta-lactam antibiotics. In therapeutic settings, it is typically combined with beta-lactamase inhibitors, predominantly clavulanate, to overcome resistance mechanisms [53]. In contrast to gentamicin and ciprofloxacin, amoxicillin did not alter any measured respiratory parameters. This supports the notion that toxic effects on mitochondria are not a universal feature of all antibiotics and may be specific to certain structural classes or mechanisms of action [46,54].

## 4. Materials and Methods

Antimycin A (cat.# A8674), ascorbate (cat.# A7631), CCCP (cat.# C2759), digitonin (cat.# D5628), DMEM (cat.# D5671), DPBS (cat.# D8537), glutamate (cat.# G1626), KH_2_PO_4_ (cat.# P5655), malate (cat.# M1000), MgCl_2_ 1 M (cat.# M1028), pyruvate (cat.# P2256), rotenone (cat.# R8875), safranin O (cat.# S2255 in the following only referred to as safranin), succinate (cat.# 74 S2378), TMPD (cat.# T3134) and trypsin-EDTA (cat.# T3924) were purchased from Sigma Aldrich, St. Louis, MA, USA. ADP (+Mg ions, cat.# 72696-48-1) was purchased from Calbiochem, San Diego, CA, USA, trypan blue (cat.# T10282) was obtained from Invitrogen, Carlsbad, CA, USA/Thermo Fisher Scientific, Waltham, MA, USA and Rhodamine 123 was purchased from Life Technologies, Carlsbad, CA, USA (cat.# R302). Gentamicin sulfate was received from AdipoGen Lifescience, San Diego, CA, USA (cat.# AG-CN2-0066-G001) and ciprofloxacin hydrochloride was acquired by Cayman Chemical company, Ann Arbor, MI, USA (cat.#14286). MiR05-Kit was supplied by Oroboros Instruments, Innsbruck, Austria (cat.# 60101-01), from which mitochondrial respiration medium Mir05 was prepared, containing 0.5 mM EGTA, 3 mM MgCl2, 60 mM lactobionic acid, 20 mM taurine, 10 mM KH2PO4, 20 mM HEPES, 110 mM sucrose, 1 g/L BSA, and the pH adjusted with KOH to 7.1.

Malate, succinate, cytochrome *c*, ascorbate, safranin, and gentamicin were dissolved in deionized H_2_O; digitonin and Rhodamine 123 were dissolved in DMSO. Antimycin A, CCCP, oligomycin, and rotenone were dissolved in ethanol p.a; amoxicillin and ciprofloxacin were dissolved in 0.1 N HCl. All solutions were aliquoted and stored at −20 °C, except pyruvate, which was prepared freshly in deionized H_2_O on the day of each experiment and safranin and ciprofloxacin, which were stored at 4 °C.

HEK 293T cells (ATCC^®^ CRL-3216™, RRID:CVCL_0063) were obtained from the American Type Culture Collection through LGC Standards (Wesel, Germany).

**Sample preparation.** Fresh and cryopreserved HEK 293T cells were provided by Oroboros Instruments GmbH in collaboration with Eduard Stefan (Institute of Biochemistry, Center for Chemistry and Biomedicine, University of Innsbruck, Innsbruck, Austria). Cells were cultured in DMEM, supplemented with 2 mM glutamine, 10% FCS, and 1% penicillin and streptomycin and maintained at 37 °C, in 5% CO_2_ and 98% humidity. For cryopreservation, cells were harvested and resuspended in a freezing solution containing 90% FCS and 10% DMSO with a cell concentration of approximately 7·10^7^ x/mL, aliquoted in cryogenic storage vials (1.2·10^7^ to 1.6·10^7^ cells per vial), and frozen at −80 °C.

Preparation of cryopreserved cells for experiments: 750 µL (if one vial of cryopreserved cells was used) or 500 µL (if two vials of cryopreserved cells were used) of mitochondrial respiration medium (MiR05) were added to each vial and pooled if more than one vial was used.

Preparation of fresh cells for experiments: adherent cells were washed once with phosphate-buffered saline (PBS) and detached by trypsinization. Cells were resuspended in DMEM and settled down via centrifugation (200× *g*, 10 min, 4 °C). For resuspension, 1 mL DMEM was added and cells separated by pipetting up and down. A second centrifugation step was used to resuspend the cells in respiration medium MiR05 (final volume 500 µL).

For counting, cells were diluted 1:10 in PBS and thereafter mixed 1:1 with trypan blue, which was also used for cell viability determination prior to the experiment. Cell count concentration was determined by Countess II AMQAX1000 Automated Cell Counter (Thermo Life Technologies, Carlsbad, CA, USA). Cell viability ranged from 70 to 95%.

**High-resolution respirometry.** Oxygen consumption rates of living and permeabilized cells were measured by HRR in the Oroboros Bioenergetics Platform following the SUIT protocol, SUIT-008-D025, and using the software DatLab 7.4 (instrument, protocol, and software by Oroboros Instruments, Innsbruck, Austria). Four Oroboros, each with two independent 2.0 mL chambers, were operated in parallel. Continuous data recording was set at 2 s time intervals and temperature was set to 37 °C. Standardized calibrations and instrumental O_2_ background tests were performed [55]. All experiments were carried out in respiration medium MiR05-Kit. HEK 293T cells were added by partial volume replacement [56]. Specifically, the stopper was removed, and a volume of MiR05 equivalent to the cell suspension containing 2.07·10⁶ cells was withdrawn from the chamber. Subsequently, 2.07·10^6^ cells were added to each chamber and stirred for 1 min. Finally, the stoppers were fully inserted, and any excess medium was siphoned off from the stopper receptacle. The concentrations of chemicals given below refer to the initial experimental concentration, i.e., the concentration in the chamber immediately after addition. These concentrations were slightly diluted over the course of the experiment due to subsequent titrations. Routine respiration was measured before the addition of 10 µg/mL digitonin. Digitonin is a mild detergent which selectively permeabilizes the plasma membrane due to its high cholesterol content, while mitochondrial membranes, which have lower cholesterol levels, are only affected at higher concentrations of digitonin. The optimum digitonin concentration was determined in previous experiments. Upon permeabilization of the plasma membrane, intracellular substrates are diluted, and respiration depends on residual endogenous substrates (*ren*). By adding 5 mM pyruvate and 2 mM malate, leak respiration in the NADH-linked (N) pathway was initiated. Then, 2 mM ADP was added to measure oxidative phosphorylation (OXPHOS) capacity. Possible mitochondrial outer membrane damage was determined by the addition of 10 µM cytochrome *c* (cyt *c*). Damage of the outer membrane provokes a loss of cyt *c* from the intermembrane space wherefore cyt *c* becomes a limiting factor for respiration. In this case, the addition of cyt *c* to the chamber increases respiration. OXPHOS capacity of the N-pathway was measured after the addition of 10 mM glutamate. The addition of 10 mM succinate supported electron flow into the Q-junction via CII to measure OXPHOS capacity in the convergent NADH and succinate-linked pathway (NS-pathway). The uncoupler CCCP was titrated stepwise (0.5 µM steps) up to an optimum concentration to induce maximum oxygen flux as a measure of NS-linked electron transfer (ET) capacity. ET capacity in the succinate-linked pathway (S-pathway) was determined upon adding 0.5 µM rotenone, an inhibitor of Complex I. Residual oxygen consumption *rox* was determined after adding the CIII inhibitor antimycin A (2.5 µM).

To determine CIV activity, 2 mM ascorbate and 0.5 mM TMPD were sequentially added to the chambers and incubated for 20 min with an open position of the stoppers. Ascorbate maintains TMPD in a steady redox state which in turn reduces cytochrome *c*. After closing, respiration was measured for 5 min, whereupon 200 mM azide, a CIV inhibitor, was added to determine chemical background oxygen flux. CIV activity was calculated by subtracting the chemical background, i.e., the oxygen flux obtained immediately after the addition of azide, from the total oxygen flux obtained immediately before the addition of azide. Relative CIV activity was obtained by normalization for the respective solvent control.

Antibiotics were titrated into the chambers at the following coupling and pathway control states: N-pathway leak state (N*_L_*), N-pathway or convergent NS-pathways OXPHOS state (N*_P_* or NS*_P_*), and convergent NS-pathway or S-pathway ET state (NS*_E_* or S*_E_*, see Table 1). The antibiotics were administered using a dose-titration schedule, outlined below, and were discontinued if a predetermined effect was achieved: gentamicin (0.1, 1.0, 2.0, and 4 mg/mL), ciprofloxacin (0.01, 0.1, 1.0, and 2 mg/mL), and amoxicillin (0.01, 0.1, 1.0, and 2 mg/mL). Solvent-matched controls were run in parallel (see above). A reported gentamicin accumulation in the kidney of 540 µg/g (ca. 1.13 mM) served as a reference of gentamicin-caused nephrotoxicity [41]. To explore this effect further and to determine if there were direct effects without prior incubation, we utilized a concentration approximately eight times greater as highest titration (4 mg/mL, corresponding to 8.37 mM). Since ciprofloxacin doses and also serum peaks under therapy (2.4–5 µg/mL [57]) are usually lower than gentamicin and amoxicillin (5–12 µg/mL [58,59]), 10 µg/mL was used as the starting point for the titrations, and the titrated up to 2 mg/mL maximum. Each experiment was repeated at least three times. Data analysis of O_2_ consumption was performed using the templates provided with the software DatLab 7.4.

**Mitochondrial membrane potential.** To measure the mitochondrial membrane potential (mtMP), the fluorescent dye safranin (λex 495 nm, λem 587 nm) was used. Safranin is a lipophilic cation, accumulating in mitochondria according to the electric potential difference between the anodic and cathodic matrix phase in energized mitochondria. Accumulation causes quenching, resulting in a fluorescence decay, which is proportional to the mtMP [60].

Measurements were performed simultaneously with oxygen consumption using smart Fluo-Sensors Blue, equipped with Filter Set Saf (Oroboros Instruments). For safranin, fluorescence intensity was set in DatLab 7.4 to 220 and the gain to 1000. Prior to all experiments, the chemical background interference was determined for the dyes with substrates and inhibitors. An initial calibration was performed before each measurement by titrating the dye in four steps to experimental concentrations of 0.5 to 2 µM (Safranin). Thereupon, 2.07·10^6^ cells were added to each chamber and routine respiration was measured. Titration steps were performed similarly to respiration measurement (using SUIT-008-D025), except for cytochrome *c*, ascorbate, and TMPD as they affect the fluorescence signal. Mitochondrial membrane potential was estimated in permeabilized cells according to the MiPNet24.08 Safranin Analysis and using the respective excel template, with the assumptions that 1·10^6^ HEK 293T cells contain approx. 14 µg of mitochondria and ca. 200 µg total protein [61].

Antibiotics were titrated to one chamber in the leak state (after permeabilization by digitonin and addition of pyruvate and malate). In the other chamber, the same amount of solvent was added as a control. The solvent of ciprofloxacin and amoxicillin affected the fluorescent signal of safranin. Therefore, only the measurement using gentamicin was repeated and used for analysis.

**Statistics.** Throughout this study, *N* is defined as the number of biological replicates and data are presented as mean ± standard deviation. Comparisons between two groups were made using the Mann–Whitney test. Differences between more than two groups with a single variable were determined using one-way ANOVA (Kruskal–Wallis), followed by Dunn’s post hoc test for multiple comparisons. When multiple groups with more than one variable were analyzed, two-way ANOVA followed by Tukey’s post hoc test was used. GraphPad Prism 8 (GraphPad Software, Inc., San Diego, CA, USA) was used for all statistical analyses. All statistical details regarding significance and *N* are given in the legends of the main and supplementary figures. *p* values are shown in the corresponding graphs.

## 5. Conclusions

This present study revealed diverse direct, dose- and time-dependent toxic effects of gentamicin and ciprofloxacin on mitochondria in permeabilized HEK 293T cells, supporting previous findings from in vitro and in vivo studies using alternate methods and technologies. This demonstrates that HRR represents an excellent methodological approach for detecting potential direct toxic effects during early stages of drug development. Most relevant is the observed substantial antibiotic-induced inhibition of the oxidative phosphorylation and reduction of ET capacity. Clearly, upon increased workload or stress situations, this would limit the bioenergetic power of affected cells, thereby plausibly paving the way to, e.g., nephrotoxicity. Indeed, our findings at the level of permeabilized cells using HRR align with observations from long-term studies in that respect.

Although the results cannot be directly extrapolated to in vivo conditions, analyses obtained from HRR on mitochondrial preparations serve as valuable indicators of toxicity and can guide the design of more effective in vivo studies or clinical trials. Notably, the assessment of direct mitochondrio-toxic effects in kidney cells justifies further risk assessments in experimental models or in patients with increased renal susceptibility.

## Figures and Tables

**Table 1:** Pathway and coupling control states (Gnaiger, 2020) assessed in SUIT-008-D025. The corresponding rates were corrected for residual oxygen consumption (*rox*).

Abbreviation	Description
N*_L_*	N-pathway in leak state
N*_P_*	N-pathway in OXPHOS state
NS*_P_*	convergent NS-pathway in OXPHOS state
NS*_E_*	convergent NS-pathway in ET state
S*_E_*	S-pathway in the presence of rotenone in ET state

## Data Availability

The original contributions presented in this study are included in the article/Supplementary Material. Further inquiries can be directed to the corresponding author(s).

## Supplementary Materials

The following supporting information can be downloaded at: https://www.mdpi.com/article/10.3390/ijms26115379/s1.

## Abbreviations

The following abbreviations are used in this manuscript:

ADP	Adenosindiphosphate
ATP	Adenosintriphosphate
BSA	Bovine serum albumin
CCCP	Carbonyl-cyanide 3-chlorophenylhydrazone
CIV	Complex IV
cyt *c*	Cytochrome *c*
DMSO	Dimethylsulfoxide
EDTA	Ethylenediaminetetraacetic acid
EGTA	Ethylene glycol-bis(β-aminoethyl ether)-N,N,N′,N′-tetraacetic acid
ET	Electron transfer
FDA	Food and Drug Administation
HEPES	4-(2-hydroxyethyl)-1-piperazineethanesulfonic acid
HRR	High-resolution respirometry
MiR05	Mitochondrial respiration medium
mt	Mitochondria(l)
mtMP	Mitochondrial membrane potential
N-pathway	NADH-linked pathway
N*_L_*	Leak respiration in the N-pathway
N*_P_*	N-pathway OXPHOS capacity
NS*_E_*	NS-pathway ET capacity
NS*_P_*	NS-pathway OXPHOS capacity
OXPHOS	Oxidatve phosphorylation
PBS	Phosphate buffered saline
*ren*	Residual endogenous substrates
ROS	Reactive oxygen species
*rox*	Residual oxygen consumption
S-pathway	Succinate-linked pathway
S*_E_*	S-pathway ET capacity
SUIT	Substrate-Uncoupler-Inhibitor titration
TMPD	N,N,N′,N′-Tetramethyl-p-phenylenediamine dihydrochloride
