# Supplementary material for: Direct Effects of Clinically Relevant Antibiotics on Mitochondrial Respiration
Source: Int J Mol Sci. 2025 Jun 4;26(11):5379. doi: 10.3390/ijms26115379 (PMC12155152; doi:10.3390/ijms26115379)
Supplement: Supplementary file 1 [file ijms-26-05379-s001.zip › ijms-3662206-supplementary.pdf]

International Journal of
Molecular Sciences

MDPI

Supplements

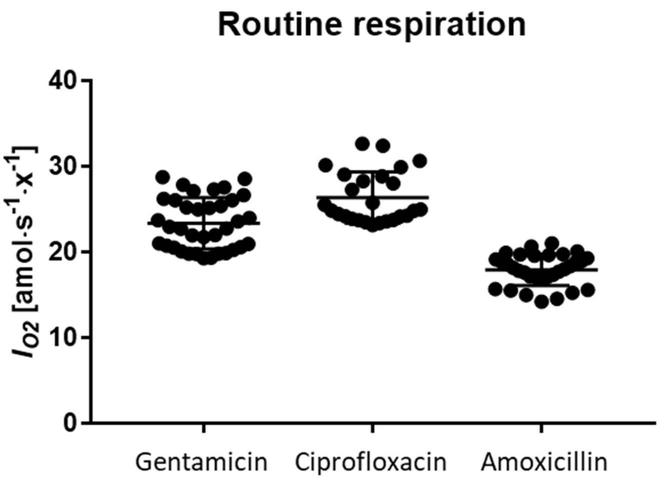

Figure S1: Routine respiration of HEK 293T cells was assessed in the beginning of each experiment, *N*=23, with n=4-8 for each biological replicate.

A slightly lower routine respiration was observed in the experiments with amoxicillin. This difference may be due to the fact that these experiments were performed at a later time point. The values are shown in Table 1S.

Table S1: Routine respiration given as specific flow [amol*s^{-1}*x^{-1}] raw data.

Gentamicin	Ciprofloxacin	Amoxicillin
22.94	24.19	18.84
21.96	24.97	17.71
19.35	32.64	14.54
20.94	32.43	15.58
28.55	30.65	19.91
27.54	30.16	19.58
26.21	23.37	18.61
26.06	24.83	18.86
21.74	25.75	17.13
21.01	25.52	17.04
23.71	28.84	14.99
21.97	28.03	15.53
26.64	23.76	19.64
28.77	23.15	20.65
27.85	23.87	19.27
25.39	23.48	17.83
20.58	29.92	18.01
19.80	28.29	17.09
19.78	24.24	17.63
19.83	24.44	15.26

22.73	23.71	21.01
22.71	24.84	19.72
27.12	29.06	18.94
27.33	27.29	19.13
24.97	24.14	17.34
25.17	23.58	16.99
20.25		14.23
19.28		15.68
20.77		19.75
20.50		20.08
26.07		18.36
25.21		18.17
23.98		
23.60		
19.88		
20.09		
22.94		
21.96		

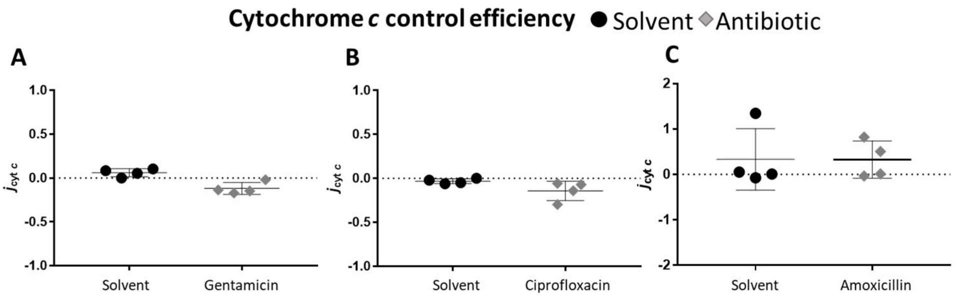

Figure S2: Effect of the antibiotics on the integrity of the outer mitochondrial membrane. Titration of the antibiotics (diamonds) in leak state did not increase the cytochrome *c* control efficiency significantly compared to the respective solvent control (circles), which indicates an intact outer mitochondrial membrane (A-C). Sequence of respiratory states (titrations and rates): see figure 1.

The effect of the antibiotics upon addition in the electron transfer state ET (NS_E). Antibiotics were titrated to permeabilized cells after the addition of substrates that fuel both the N- and S-pathway and after uncoupling the system. In this way, any immediate effects on mitochondrial electron transfer capacity could be specifically assessed.

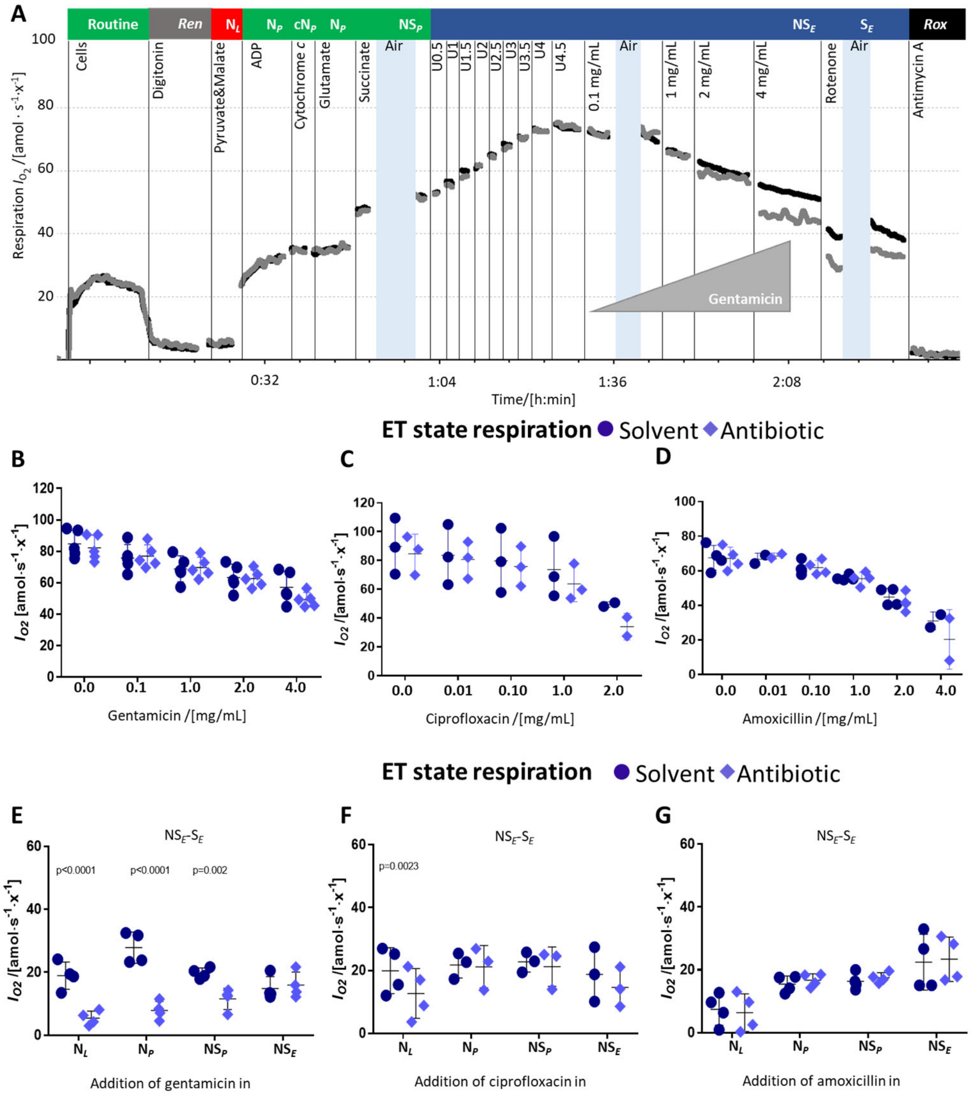

Figure S3: Effect of antibiotics on NS- and S-linked ET capacity which were progressively inhibited at an optimum uncoupler concentration. Gentamicin and ciprofloxacin slightly inhibit the electron transfer system of HEK 293T cell mitochondria. A) Representative trace of O2 flow per cell [amol·s^{-1}·x^{-1}] using the the protocol SUIT-008_D025 (37°C, MiR05) with titration of gentamicin (grey trace) or its solvent (black trace) in ET state. Sequence of respiratory states (titrations and rates): see figure 1. (B-D) O_2 flow per cell [amol O_2·s^{-1}·x^{-1}] in ET upon titration of gentamicin (B), ciprofloxacin (C), or amoxicillin (D) (blue diamond) or respective solvent control (blue circle). *Gentamicin shows a time-dependent inhibitory effect on the N- pathway.* (E-G) Delta in respiration between NS_E and S_E. Antibiotic (diamonds) or solvent (circles) were added in the different respiratory states, each data point represents a biological replicate (*N*=3-4). Concentration up to 4 mg/mL for gentamicin and 2 mg/mL for ciprofloxacin and amoxicillin. Medians are depicted as stripes.

The antibiotics or respective solvent controls were added to the chambers in four steps. A continuous decrease in respiration was observed after each titration of both the antibiotics and the solvent control. After titration of the highest concentration, a slightly more pronounced decrease in respiration was observed with the antibiotics (Figure 2S B-D) compared to the respective solvent control. The overall decrease in respiration over time is most likely due to the inhibitory effect of the uncoupler.

When comparing the delta of respiration in S_E and NS_E, between gentamicin and solvent control treated cells, the highest difference can be observed when the antibiotic is titrated in leak, this is in the beginning of the protocol. Whereas the delta of the solvent control treated cells is relatively stable, independent of the time point of solvent titration,

the delta of the gentamicin treated cells increases over time, i.e. upon titration of the antibiotic at a later time point in the protocol (figure 3SE). The increase in the delta is mainly due to an increase in respiration in NS_E and not to a decrease in respiration in S_E supporting the time dependent inhibition of the N-pathway. Ciprofloxacin might have a similar but less pronounced effect whereas no clear differences can be observed for amoxicillin (figure 3SF, G).

Table S2: Mean values of the specific flow [amol*s^{-1}*x^{-1}] as depicted in figure 3S (Effect of antibiotics on NS- and S-linked ET capacity).

	**Solvent**	**Solvent STD**	**Gentamicin**	**Gentamicin STD**
**N_L (PM)**	4.99	1.00	6.03	1.24
**N_P (PM)**	34.82	3.47	38.40	4.97
**N_P (PMG)**	37.67	2.90	41.27	5.75
**NS_P (PMGS))**	58.04	4.44	62.74	7.98
**NS_E (PMGS)**	81.71	7.70	84.74	11.03
**Gentamicin 0.1 mg/mL**	74.93	8.29	80.79	10.47
**Gentamicin 1 mg/mL**	68.23	7.76	75.85	11.24
**Gentamicin 2 mg/mL**	62.91	7.87	63.82	10.22
**Gentamicin 4 mg/mL**	57.38	9.19	48.59	21.20
**S_E (S)**	42.55	7.87	33.99	17.63

	**Solvent**	**Solvent STD**	**Ciprofloxacin**	**Ciprofloxacin STD**
**N_L (PM)**	4.98	2.57	6.03	1.24
**N_P (PM)**	30.95	15.35	38.40	4.97
**N_P (PMG)**	33.14	16.34	41.27	5.75
**NS_P (PMGS))**	49.95	24.76	62.74	7.98
**NS_E (PMGS)**	71.20	34.94	84.74	11.03
**Ciprofloxacin 0.01 mg/mL**	66.88	32.50	80.79	10.47
**Ciprofloxacin 0.1 mg/mL**	64.37	31.12	75.85	11.24
**Ciprofloxacin 1 mg/mL**	59.48	28.76	63.82	10.22
**Ciprofloxacin 2 mg/mL**	54.20	26.77	48.59	21.20
**S_E (S)**	38.75	19.36	33.99	17.63

	**Solvent**	**Solvent STD**	**Amoxicillin**	**Amoxicillin STD**
**N_L (PM)**	4.38	0.50	4.10	0.87
**N_P (PM)**	27.47	1.73	26.83	2.10
**N_P (PMG)**	28.97	2.07	27.88	2.66
**NS_P (PMGS))**	46.25	2.57	47.08	3.46
**NS_E (PMGS)**	67.54	6.24	67.12	5.72
**Amoxicillin 0.01 mg/mL**	66.70	2.46	68.56	1.23

**Amoxicillin 0.1 mg/mL**	61.45	3.46	61.88	3.53
**Amoxicillin 1 mg/mL**	55.92	1.38	55.43	3.22
**Amoxicillin 2 mg/mL**	44.83	4.34	41.90	4.44
**Amoxicillin 4 mg/mL**	31.06	3.68	20.38	12.18
**S*E* (S)**	22.39	3.84	18.52	7.60

Table S3: Mean values of the specific flow [amol*s^{-1}*x^{-1}] depicted in figure 1 (Effect of antibiotics on N-linked leak respiration and subsequent pathway and coupling control states).

	**Solvent**	**Solvent STD**	**Gentamicin**	**Gentamicin STD**
**N*L* (PM)**	4.89	0.47	5.68	0.80
**Gentamicin 0.1 mg/mL**	4.84	0.23	5.50	0.96
**Gentamicin 1 mg/mL**	5.15	0.42	8.05	1.23
**Gentamicin 2 mg/mL**	4.88	0.05	16.90	2.65
**Gentamicin 4 mg/mL**	4.52	0.18	11.86	0.74
**N*P* (PM)**	26.08	2.11	13.82	0.69
**N*P* (PMG)**	27.29	1.14	11.88	0.94
**NS*P* (PMGS)**	50.85	3.47	33.31	2.60
**NS*E* (PMGS)**	66.96	3.55	34.14	4.21
**S*E* (S)**	48.04	5.79	28.70	2.58
	**Solvent**	**Solvent STD**	**Ciprofloxacin**	**Ciprofloxacin STD**
**N*L* (PM)**	6.54	1.55	6.31	1.64
**Ciprofloxacin 0.01 mg/mL**	6.56	1.00	6.30	1.57
**Ciprofloxacin 0.1 mg/mL**	6.73	1.14	6.02	1.49
**Ciprofloxacin 1 mg/mL**	6.62	1.04	5.70	1.36
**Ciprofloxacin 2 mg/mL**	6.39	0.97	13.17	4.86
**N*P* (PM)**	6.23	1.15	14.53	3.78
**N*P* (PMG)**	30.05	6.47	16.68	6.67
**NS*P* (PMGS)**	54.36	9.23	37.28	11.09
**NS*E* (PMGS)**	69.26	14.23	40.00	13.70
**S*E* (S)**	6.54	1.55	6.31	1.64
	**Solvent**	**Solvent STD**	**Amoxicillin**	**Amoxicillin STD**
**N*L* (PM)**	4.03	0.54	3.71	0.74
**Amoxicillin 0.01 mg/mL**	3.88	0.00	4.70	0.00

**Amoxicillin 0.1 mg/mL**	3.99	0.21	3.37	0.54
**Amoxicillin 1 mg/mL**	3.73	0.49	3.29	0.53
**Amoxicillin 2 mg/mL**	3.90	0.31	3.44	0.76
**Amoxicillin 4 mg/mL**	3.84	0.96	2.07	1.16
$N_{P\,(PM)}$	19.43	4.44	15.58	9.44
$N_{P\,(PMG)}$	17.17	3.29	14.50	7.23
$NS_{P\,(PMGS)}$)	30.18	5.07	23.88	11.73
$NS_{E\,(PMGS)}$	35.77	8.77	28.43	14.49
$S_{E\,(S)}$	26.18	6.36	20.00	10.10

Table S4: Mean values of the specific flow [amol*s^{-1}*x^{-1}] depicted in figure 2 (Effect of antibiotics on N-linked OXPHOS capacity and subsequent pathway and coupling control states).

	**Solvent**	**Solvent STD**	**Gentamicin**	**Gentamicin STD**
$N_{L\,(PM)}$	5.54	0.94	5.12	0.87
$N_{P\,(PM)}$	36.62	4.40	35.83	4.26
$N_{P\,(PMG)}$	39.35	4.26	38.50	3.57
**Gentamicin 0.1 mg/mL**	39.46	3.99	38.54	3.95
**Gentamicin 1 mg/mL**	38.98	3.86	38.11	3.83
**Gentamicin 2 mg/mL**	38.02	3.87	39.25	3.49
**Gentamicin 4 mg/mL**	35.21	3.27	22.92	2.92
$NS_{P\,(PMGS)}$)	58.46	5.82	35.92	4.84
$NS_{E\,(PMGS)}$	77.59	8.30	37.29	7.02
$S_{E\,(S)}$	49.77	6.96	29.41	5.00
	**Solvent**	**Solvent STD**	**Ciprofloxacin**	**Ciprofloxacin STD**
$N_{L\,(PM)}$	6.76	0.77	6.50	1.27
$N_{P\,(PM)}$	40.08	5.99	41.44	6.45
$N_{P\,(PMG)}$	41.54	7.18	43.66	7.71
**Ciprofloxacin 0.01 mg/mL**	42.14	6.99	43.58	7.64
**Ciprofloxacin 0.1 mg/mL**	41.74	7.09	37.49	8.17
**Ciprofloxacin 1 mg/mL**	45.74	7.97	38.23	9.88
$NS_{P\,(PMGS)}$)	59.83	7.28	54.74	7.28
$NS_{E\,(PMGS)}$	80.30	10.17	73.76	10.57

**S_E (S)**	58.51	9.57	52.56	11.19

	**Solvent**	**Solvent STD**	**Amoxicillin**	**Amoxicillin STD**
**N_L (PM)**	4.04	0.69	3.99	0.68
**N_P (PM)**	28.33	1.71	27.88	2.87
**N_P (PMG)**	29.72	1.82	28.84	3.46
**Amoxicillin 0.01 mg/mL**	30.66	1.39	29.96	2.83
**Amoxicillin 0.1 mg/mL**	28.95	1.90	28.67	3.09
**Amoxicillin 1 mg/mL**	27.02	1.50	26.29	3.00
**Amoxicillin 2 mg/mL**	23.43	1.21	22.70	2.56
**Amoxicillin 4 mg/mL**	21.37	1.15	19.87	0.04
**NS_P (PMGS))**	38.81	2.74	36.81	3.41
**NS_E (PMGS)**	49.27	3.00	45.26	3.75
**S_E (S)**	33.79	1.02	28.52	2.47

Table S5: Mean values of the specific flow [amol*s^{-1}*x^{-1}] depicted in figure 3 (Effect of antibiotics on NS-linked OXPHOS capacity and subsequent pathway and coupling control states).

	**Solvent**	**Solvent STD**	**Gentamicin**	**Gentamicin STD**
**N_L (PM)**	5.94	0.55	5.04	1.06
**N_P (PM)**	36.08	4.06	29.80	11.79
**N_P (PMG)**	38.93	3.53	38.15	3.14
**NS_P (PMGS))**	56.95	6.29	56.48	6.06
**Gentamicin 0.1 mg/mL**	57.39	5.77	56.20	5.14
**Gentamicin 1 mg/mL**	57.89	5.84	54.84	5.02
**Gentamicin 2 mg/mL**	56.18	4.27	52.89	4.46
**Gentamicin 4 mg/mL**	56.00	2.29	38.72	2.01
**NS_E (PMGS)**	73.01	2.25	41.64	5.26
**S_E (S)**	53.31	1.41	30.08	2.99

	**Solvent**	**Solvent STD**	**Ciprofloxacin**	**Ciprofloxacin STD**
**N_L (PM)**	6.86	1.54	6.26	1.10
**N_P (PM)**	40.78	6.38	39.59	6.43
**N_P (PMG)**	43.82	6.68	42.55	6.90
**NS_P (PMGS))**	65.79	10.88	64.63	11.13
**Ciprofloxacin 0.01 mg/mL**	66.86	10.69	64.27	10.34
**Ciprofloxacin 0.1 mg/mL**	66.22	10.19	63.00	9.92

**Ciprofloxacin 1 mg/mL**	62.97	9.45	51.11	8.15
**NS$_{E\ (PMGS)}$**	83.72	11.75	69.08	11.56
**S$_{E\ (S)}$**	61.02	9.77	47.87	9.39
	**Solvent**	**Solvent STD**	**Amoxicillin**	**Amoxicillin STD**
**N$_{L\ (PM)}$**	4.02	0.83	4.31	0.45
**N$_{P\ (PM)}$**	28.48	2.32	29.09	1.29
**N$_{P\ (PMG)}$**	29.10	2.98	30.23	1.15
**NS$_{P\ (PMGS}$)**	50.51	3.48	50.36	0.43
**Amoxicillin 0.01 mg/mL**	47.15	4.24	48.51	1.60
**Amoxicillin 0.1 mg/mL**	43.86	3.86	44.18	1.27
**Amoxicillin 1 mg/mL**	38.65	3.26	38.16	1.43
**Amoxicillin 2 mg/mL**	35.65	0.07	36.31	3.93
**Amoxicillin 4 mg/mL**	49.10	4.52	50.37	1.81
**NS$_{E\ (PMGS)}$**	49.89	4.01	47.43	4.66
**S$_{E\ (S)}$**	33.53	2.19	29.92	4.24

Table S6: Mean values of the specific flow [amol*s^{-1}*x^{-1}] depicted in figure 4 (Time dependent effect of gentamicin on OXPHOS and ET capacity).

**OXPHOS respiration N$_P$**				
**Addition in**	**Solvent**	**Solvent STD**	**Gentamicin**	**Gentamicin STD**
**N$_{L\ (PM)}$**	27.29	1.37	10.86	1.15
**N$_{P\ (PMG)}$**	35.21	3.27	22.92	2.92
**Addition in**	**Solvent**	**Solvent STD**	**Ciprofloxacin**	**Ciprofloxacin STD**
**N$_{L\ (PM)}$**	32.03	6.33	19.63	4.95
**N$_{P\ (PMG)}$**	45.60	7.97	38.26	9.97
**Addition in**	**Solvent**	**Solvent STD**	**Amoxicillin**	**Amoxicillin STD**
**N$_{L\ (PM)}$**	17.17	3.29	15.81	7.28
**N$_{P\ (PMG)}$**	23.43	1.21	22.70	2.56
**OXPHOS respiration NS$_P$**				
**Addition in**	**Solvent**	**Solvent STD**	**Gentamicin**	**Gentamicin STD**
**N$_{L\ (PM)}$**	50.85	3.82	32.29	2.65
**N$_{P\ (PMG)}$**	58.46	5.82	35.92	4.84
**NS$_{P\ (PMGS}$)**	56.00	2.29	38.72	2.01

Addition in	Solvent	Solvent STD	Ciprofloxacin	Ciprofloxacin STD
N_L (PM)	54.99	10.59	40.61	10.94
N_P (PMG)	59.83	7.28	54.74	7.28
NS_P (PMGS)	62.97	9.45	51.11	8.15

Addition in	Solvent	Solvent STD	Amoxicillin	Amoxicillin STD
N_L (PM)	30.18	5.07	23.88	11.73
N_P (PMG)	37.46	1.63	34.91	1.03
NS_P (PMGS)	37.04	1.96	37.69	1.36

ET respiration NS_E

Addition in	Solvent	Solvent STD	Gentamicin	Gentamicin STD
N_L (PM)	66.96	3.89	33.12	4.15
N_P (PMG)	77.59	8.30	37.29	7.02
NS_P (PMGS)	73.01	2.25	41.64	5.26
NS_E (PMGS)	57.38	9.19	47.76	4.56

Addition in	Solvent	Solvent STD	Ciprofloxacin	Ciprofloxacin STD
N_L (PM)	71.29	15.93	44.89	12.44
N_P (PMG)	80.30	10.17	73.76	10.57
NS_P (PMGS)	83.72	11.75	69.08	11.56
NS_E (PMGS)	65.01	22.08	48.59	21.20

Addition in	Solvent	Solvent STD	Amoxicillin	Amoxicillin STD
N_L (PM)	35.77	8.77	28.43	14.49
N_P (PMG)	48.05	2.45	43.37	2.12
NS_P (PMGS)	47.86	2.24	46.48	5.03
NS_E (PMGS)	46.23	4.16	41.99	5.13

ET respiration S_E

Addition in	Solvent	Solvent STD	Gentamicin	Gentamicin STD
N_L (PM)	48.04	6.23	27.68	2.41
N_P (PMG)	49.77	6.96	29.41	5.00
NS_P (PMGS)	53.31	1.41	30.08	2.99
NS_E (PMGS)	42.55	7.87	31.85	3.10

Addition in	Solvent	Solvent STD	Ciprofloxacin	Ciprofloxacin STD
N_L (PM)	48.74	11.00	29.20	7.75
N_P (PMG)	58.51	9.57	52.56	11.19
NS_P (PMGS)	61.02	9.77	47.87	9.39

**NS$_{E}$ (PMGS)**	46.23	16.59	33.99	17.63

Addition in	Solvent	Solvent STD	Amoxicillin	Amoxicillin STD
**N$_{L}$ (PM)**	26.18	6.36	20.00	10.10
**N$_{P}$ (PMG)**	33.27	0.54	27.23	1.18
**NS$_{P}$ (PMGS)**	32.71	1.93	29.03	4.57
**NS$_{E}$ (PMGS)**	21.32	3.89	16.78	8.05

Table S7: Mean values of relative complex IV activity data depicted in figure 5.

Relative complex IV activity				
**Addition in**	**Solvent**	**Solvent STD**	**Gentamicin**	**Gentamicin STD**
**N$_{L}$ (PM)**	100	0	57.85	5.54
**NS$_{E}$ (PMGS)**	100	0	77.09	3.44

Addition in	Solvent	Solvent STD	Ciprofloxacin	Ciprofloxacin STD
**N$_{L}$ (PM)**	100	0	102.51	3.18
**NS$_{E}$ (PMGS)**	100	0	124.94	19.07

Addition in	Solvent	Solvent STD	Amoxicillin	Amoxicillin STD
**N$_{L}$ (PM)**	100	0	86.01	5.58
**NS$_{E}$ (PMGS)**	100	0	98.40	4.52

Table S8: Mean values of mitochondrial membrane potential as depicted in figure 6.

	Solvent	Solvent STD	Gentamicin	Gentamicin STD
**N$_{L}$ (PM)**	1.00	0.00	1.00	0.00
**N$_{P}$ (PM)**	1.03	0.01	1.09	0.01
**N$_{P}$ (PMG)**	1.02	0.02	1.09	0.01
**NS$_{P}$ (PMGS)**	1.02	0.02	0.57	0.14
**Gentamicin 0.1 mg/mL**	1.00	0.02	0.23	0.16
**Gentamicin 1 mg/mL**	0.73	0.05	0.12	0.09
**Gentamicin 2 mg/mL**	0.72	0.06	0.13	0.09
**Gentamicin 4 mg/mL**	0.65	0.07	0.07	0.07
**NS$_{E}$ (PMGS)**	0.00	0.00	0.00	0.00

Disclaimer/Publisher's Note: The statements, opinions and data contained in all publications are solely those of the individual author(s) and contributor(s) and not of MDPI and/or the editor(s). MDPI and/or the editor(s) disclaim responsibility for any injury to people or property resulting from any ideas, methods, instructions or products referred to in the content.
